# Characterization and distribution of medicine vendors in 2 states in Nigeria: implications for scaling health workforce and family planning services

**DOI:** 10.1186/s12960-021-00602-2

**Published:** 2021-05-01

**Authors:** Babajide Oluseyi Daini, Emeka Okafor, Sikiru Baruwa, Oluwafunmito Adeyanju, Rodio Diallo, Jennifer Anyanti

**Affiliations:** 1 Population Council, Abuja, Nigeria; 2 IntegratE Project, Abuja, Nigeria; 3 Pharm-Access International, Abuja, Nigeria; 4 Bill and Melinda Gates Foundation, Seattle, USA; 5 Society for Family Health, Abuja, Nigeria

**Keywords:** Family planning, Health workers, Medical training, PPMVs, Stocking, Task-shifting

## Abstract

**Background:**

In 2014, Nigeria issued the task-shifting/sharing policy for essential health services, which aimed to fill the human resource gap and improve the delivery of health services across the country. This study focuses on the characteristics, spread, and family planning (FP) stocking practices of medicine vendors in Lagos and Kaduna, assessing the influence of medical training on the provision and stocking of FP services and commodities by vendors.

**Methods:**

We conducted a census of all Patent Medicines stores (PMS) followed up with a facility assessment among 10% of the mapped shops, utilizing an interviewer-administered questionnaire. Bivariate analysis was conducted using the Chi-square test, and multiple logistic regression was used to estimate the adjusted odds ratio (OR) and confidence intervals (CI) for the test of significance in the study.

**Results:**

A total of 8318 medicine shops were enumerated (76.2% urban). There were 39 shops per 100,000 population in both states on average. About half (50.9%) were manned by a medicine vendor without assistance, 25.7% claimed to provide FP services to > 2 clients per week, and 11.4% were not registered with the regulatory body or any professional association. Also, 28.2% of vendors reported formal medical training, with 56.3% of these medically trained vendors relatively new in the business, opening within the last 5 years. Vendors utilized open drug markets as the major source of supply for FP products. Medical training significantly increased the stocking of FP products and inhibited utilization of open drug markets.

**Conclusion:**

Patent and Proprietary Medicines Vendor (PPMVs) have continued to grow progressively in the last 5 years, becoming the most proximal health facility for potential clients for different health services (especially FP services) across both Northern and Southern Nigeria, now comprising a considerable mass of medically trained personnel, able to deliver high-quality health services and complement existing healthcare infrastructure, if trained. However, restrictions on services within the PPMV premise and lack of access to quality drugs and commodities have resulted in poor practices among PPMVs. There is therefore a need to identify, train, and provide innovative means of improving access to quality-assured products for this group of health workers.

## Introduction

Across several low- and middle-income countries (LMICs), national health systems have been functioning sub-optimally due to recurring challenges within the health sector, with this particularly affecting the delivery of accessible and affordable healthcare services [1]. The health system, in most cases, is synonymous with publicly owned health facilities, with several important private and non-state actors being downplayed [2]. The functional capacities of the health system in these settings have gradually weakened, having failed to recognize and maximize efforts of all parastatal primarily devoted to improving health (including organizations, institutions, structures, and resources) [1, 3].

Health workforce is an integral part of the health system and plays a critical role in achieving effective healthcare delivery. According to the World Health Organization (WHO), they are people “primarily engaged in action with the intent of enhancing health” diagnosing illnesses, healing, caring for people, monitoring health outcomes, supporting treatment adherence, providing medical information, and preventing diseases [4, 5]. They consist of physicians, nurses, midwives, dentists, pharmacists, laboratory workers, environment and public health workers, community health workers, other health workers, and health management, and support staff [6, 7]. The correlation between the availability of health workforce and positive health outcomes has been observed by several studies [1, 8].

In 2007, there was a health workforce density of 35 doctors and 86 nurses per 100,000 populations of Nigerians comparable to sub-Saharan average of 15 doctors and 72 nurses per 100,000 population; this shortage being reported in a region that contributes to a quarter of the global burden of disease [5, 9]. These figures have remained inadequate over the years, with Nigeria reporting a health workforce of 195 (doctors and nurses) per 100,000 ten years from the 2006 world health report [10]. This scenario has been linked to inadequate production and inequitable distribution across the country [3, 10, 11]. With massive historical brain drain which already has been documented to have caused setbacks in the country’s health system [12] and declining recent brain drain still being experienced in Africa (including Nigeria); especially of doctors (25%) [10] and nurses (5%) [13], the number of community pharmacists are also grossly inadequate [14] and hence the need for other cadres of staff to fill in the gap and improve healthcare (especially basic public health services).

Although not always recognized as front-line health workers, across many countries in sub-Saharan Africa (including Nigeria), medicine shops offer an alternative, when there are shortages in the health sector (including human resource shortage), and are usually the first point of care-seeking in most communities [15–18]. As reported by the National Demographic Health Survey (NDHS), nearly half (41%) of contraceptives users in Nigeria obtain their contraceptives from the private sector, with more than half of all mothers/guardians seeking care from this sector for childhood illness such as diarrhea (54.1%) and fever (57.7%) [19]. Major reasons noted for this occurrence in several studies include the absence of formalities, reduced waiting time at the medicine shop, the proximity of the outlets to clients, and the perception of clients that they will pay more at a health facility [20, 21]. Patent and Proprietary Medicine Vendors (PPMVs) are increasingly being recognized as important providers of health commodities, as interest continues to grow among policymakers and program implementers regarding their engagement in primary health care delivery in Nigeria [16]. In some settings, training drug vendors to provide high-quality basic services, such as family planning (FP) services, treatment of common childhood illnesses and malaria, may offer a cost-effective way of delivering community-based health programs [17, 20, 22]. The Pharmacists Council of Nigeria (PCN) undertakes the registration, regulation, and licensure of PPMVs, conducting orientation programs for new PPMVs, continuing education programs for existing PPMVs, inspection, and publishing the Approved Patent Medicine List (APML) [23]. Consequences of no registration include the closure of unlicensed shops, and penalization of those found to stock drugs outside the approved medicine list. Studies have, however, shown a reluctance of medicine vendors to register with the regulatory body and preferring to register with their professional associations, which also provide drug stocking support, facilitate education and training, and give business and financial assistance [18, 24, 25]. Registration is, however, limited to vendors who want to operate shops within the communities and not illegal drug hawkers or peddlers, who are prohibited from the practice of unauthorized sale of drugs in the open market, by the Counterfeit and Fake Drugs Act of 1990.

In 2014, Nigeria issued the task-shifting and task-sharing (TSTS) policy for essential health services in Nigeria, which aimed to fill the human resource gap and improve the delivery of health services across the country [26]. The policy highlighted the need to expand services to community-based personnel including medicine vendors to provide treatment, counseling, and referral for some reproductive, maternal, and child health services (including FP) [26, 27]. Previous studies have shown that different professionals may be operating within the PPMV space (even some with medical training [17, 28]. Thus, the integration of this group of private providers into the formal healthcare system could increase access to high-quality, primary health care services throughout the country [18].

In an initial study conducted in 2014, Liu et al. estimated the number of vendors in Nigeria and offered clues to drug stocking practices and how this group of healthcare workers could be better engaged to improve healthcare across the country [18]. However, it has been 4 years since the initial study and with the support recently showed to this group of health workers through the TSTS policy, there is the need to conduct further characterization and distribution of drug vendors. This study focuses on the characteristics, spread, and FP stocking practices of PPMVs in Lagos and Kaduna states of Nigeria. It also assesses the influence of medical training and other factors on the provision and stocking of FP services/commodities by medicine vendors.

## Methodology

Between February and September 2018, we conducted a census of all medicine shops in Lagos and Kaduna states. The census collected information on the basic characteristics of the shop, educational qualification of the owner, and number/type of support staff working in the shops. Geographical coordinates of each shop were also recorded using the Global Positioning System (GPS) configured into the electronic tool used for data collection, adjusted to an accuracy of 5 m. The states were pre-determined by the project implementation methodology when the project proposal was written and purposively selected because of a planned FP intervention, regional differences between northern and southern Nigeria and comparability of findings with the Lui et al. study conducted in 2014 (Lagos was one of the states chosen in that study and will show how things have changed since the study in 2014, whereas Kaduna will represent a new look and give insights about some states not represented in the 2014 study).

Data collectors worked with a list of PPMVs provided by the PCN and professional associations in the States. From the list, data collectors also snow-balled until all PPMVs in the local government areas (LGAs) were covered.

Following the census, a more robust facility assessment was conducted among a sample comprising about 10% of the entire mapped PPMV shops; these shops were randomly selected from the 14 implementation LGAs systematically selected for the project in the two states (5 rural and 8 urban). The assessment aimed to elicit additional information not collected during the census. This assessment occurred between October and December 2018 and utilized an interviewer-administered questionnaire programmed using the Census and Survey Processing System (*CSPro*) into mobile phones through the CSentry Application.

A Medicine vendor was regarded as having health (medical) training if he/she reported having qualification listed as medical doctor, nurse, midwife or pharmacist or having completed a training program as a community health extension worker or a 2/3-year clinical training program as a junior community health extension worker [18].

Major outcomes in this study included: (1) current stock of FP products, including any brand of oral contraceptives, injectable contraceptives (and DMPA-SC), emergency contraception, intrauterine contraceptive device; (2) registration with a professional association or the PCN; (3) source of supply of FP commodity (defined as whether or not the vendor bought FP commodities from the open market). Major predictors in the study included medical training of vendor, training attendance (defined as PPMV shops in which at least one person has ever attended training on FP products and services), and location of the PPMV shop (defined as rural or urban).

Other variables recorded in this study included the number of staff working in the facility, the estimated number of FP clients per day, and the length of time PPMV has been in business. We calculated the number of shops per 100,000 population in each local government area, using the 2006 national census estimates adjusted for population growth to 2017. In addition, the percentage of vendors who had health/medical training was calculated. Both parameters were shown graphically using maps culled from both states (showing LGAs).

Data were reported as frequencies and percentages to describe the various variables used in this study. Bivariate analysis was conducted using the Chi-square test. Multiple logistic regression was used to estimate the adjusted odds ratio (OR) and confidence intervals (CI) for the test of significance in the study. Statistical and spatial analyses were conducted using Stata v.14.0, and QGIS v.3.8, all tests were 2 sided and *p*-values < 0.05 were considered as statistically significant.

Two models were employed for the logistic regression; Model 1 examined the association between medical training of vendor and registration, stocking and source of FP products while controlling for state, location (urban/rural), number of staff, number of FP clients and length of time business started. Model 2 examined the association between length of time business started and registration, stocking and source of FP products while controlling for state, location, number of FP clients, and medical training of the vendor.

## Results

### Background characteristics

**Fig. 1:**
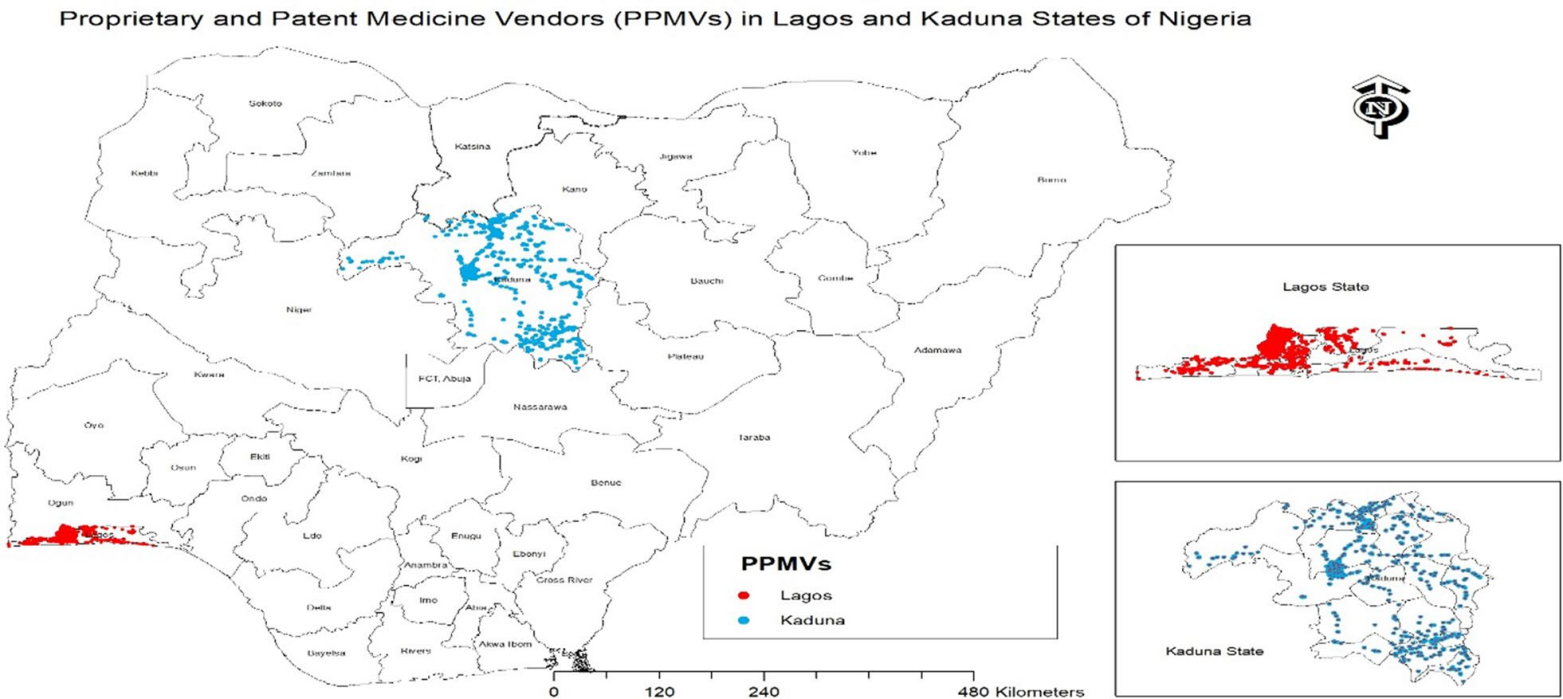
Location of PPMVs shops in 2 states. There were 2,345 shops (28.2%) in which the medicine vendor (person in charge) had some form of medical training; majority were community extension worker (14.4%), nurses (both retired and some working in health facilities) (9.6%) and other junior cadres (4.1%). Figure 2 shows the proportion of PPMVs with medical training in each of the LGAs across both states. The average percentage was much higher in the rural areas (32.2%) and in Kaduna State (37.9%) than urban areas (19.7%) and Lagos State (11.3%). About half (50.9%) of the shops were solely manned by the medicine vendor without assistance, about one-third (31.9%) had staff ranging from 1 to 3 persons and only a quarter of the shops had more than four (4) staff

We visited a total of 8318 PPMVs in the study; the majority of these were in the urban areas (76.2%) (see Fig. 1). Tables 1 and 2 show the characteristics of shops identified across the 43 local governments areas (LGAs) in both states.

**Fig. 2:**
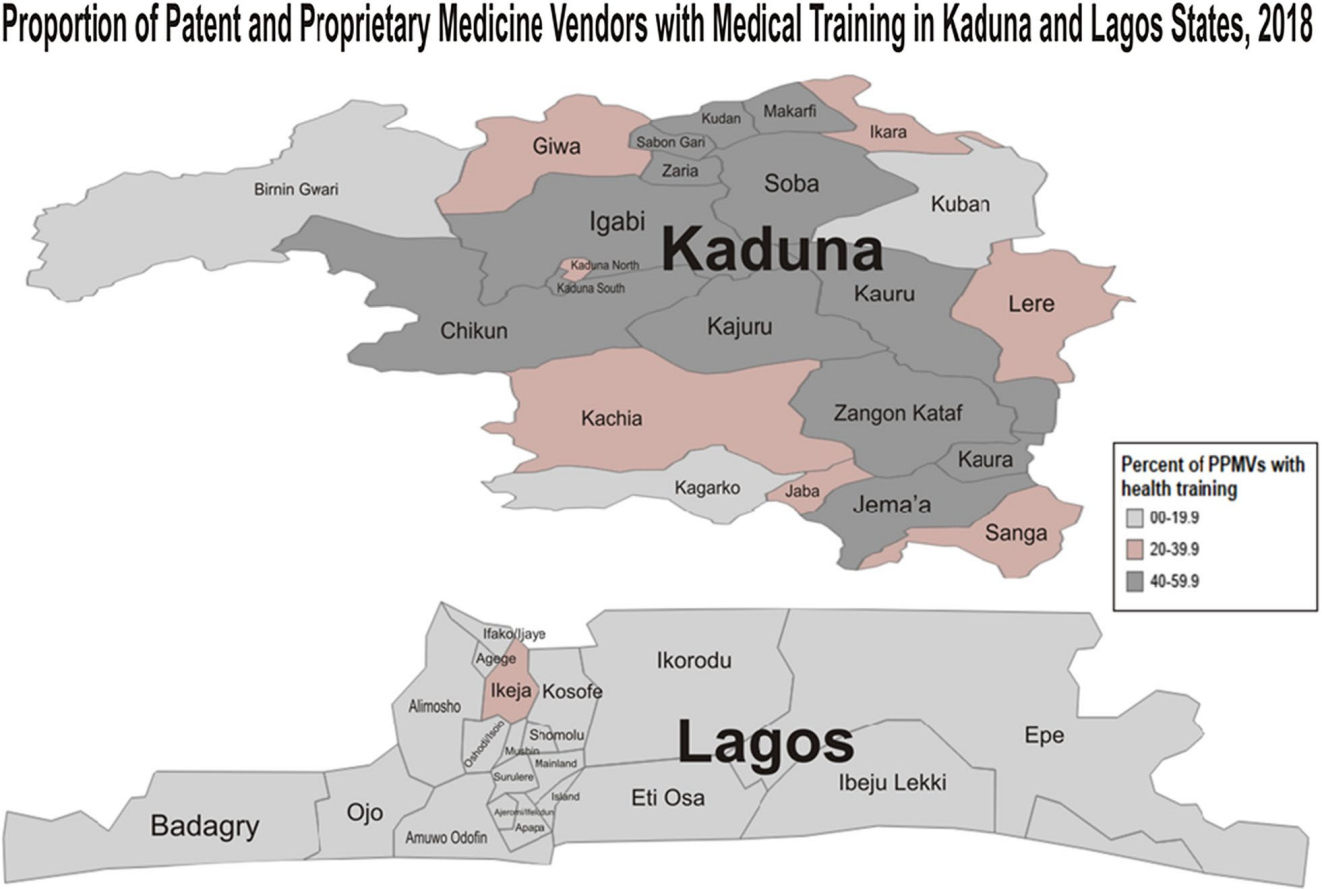
PPMVs with medical training in Kaduna and Lagos states

**Table 1:** Census of patent and proprietary medicine shops in Kaduna and Lagos states, 2018

Characteristics	Number of shops (%) (*N* = 8318)
State	
Kaduna	4572 (55.0)
Lagos	3746 (45.0)
Location	
Urban	6339 (76.2)
Rural	1979 (23.8)
Medical training of medicine vendor	
No medical training	5973 (71.8)
Community health extension worker or community health officer	1201 (14.4)
Nurse or midwife	797 (9.6)
Junior community health extension worker	133 (1.6)
Doctor	5 (0.1)
Other healthcare workers	209 (2.5)
Number of support staff*	
0	4180 (50.9)
1–3	2618 (31.9)
≥ 4	1410 (17.2)

*Data were available for only 8208 shops

**Table 2:** Summary of LGA census of PPMVs in Kaduna and Lagos states, respecitvely, 2018

LGA	Freq	PPMV/100,000 population	Proportion of PPMVs with health training	LGA	Freq	PPMV/100,000 Population	Proportion of PPMVs with health training
Kaduna State				Lagos State			
Birnin Gwari	69	20	13.0	Ajeromi/Ifelodun	112	12	4.5
Chikun	1045	205	47.1	Alimosho	999	55	8.9
Giwa	108	27	31.5	Amuwo Odofin	179	40	14.5
Igabi	666	112	46.2	Apapa	131	43	2.3
Ikara	67	25	22.4	Badagry	330	97	8.8
Jaba	42	20	33.3	Epe	73	28	9.6
Jema'a	178	46	50.6	Eti Osa	53	13	11.3
Kachia	91	27	30.8	Ibeju Lekki	148	89	19.6
Kaduna North	516	104	31.2	Ifako/Ijaye	317	52	17.0
Kaduna South	558	100	47.0	Ikeja	33	7	27.3
Kagarko	48	14	12.5	Ikorodu	327	43	16.2
Kauru	57	37	45.6	Kosofe	49	5	16.3
Kaura	47	15	57.4	Lagos Island	71	24	4.2
Kauru	65	28	52.3	Lagos Mainland	68	15	11.8
Kuban	45	12	11.1	Mushin	105	12	6.7
Kudan	66	34	48.5	Ojo	297	35	14.8
Lere	126	27	31.7	Oshodi/Isolo	151	17	6.0
Makarfi	50	25	42.0	Shomolu	100	18	8.0
Sabon Gari	272	68	41.2	Surulere	80	11	8.8
Sanga	57	28	33.3	Agege	123	19	8.9
Soba	89	22	50.6	Total	3746	32	11.3
Zangon Kataf	44	10	43.2				
Zaria	266	47	49.2				
Total	4572	46	37.9				

The number of medicine shops identified varied from 42 and 33 in Jaba and Ikeja LGAs of Kaduna and Lagos states, respectively, to 1045 and 999 identified in Chikun and Alimosho LGAs of both states.

**Fig. 3:**
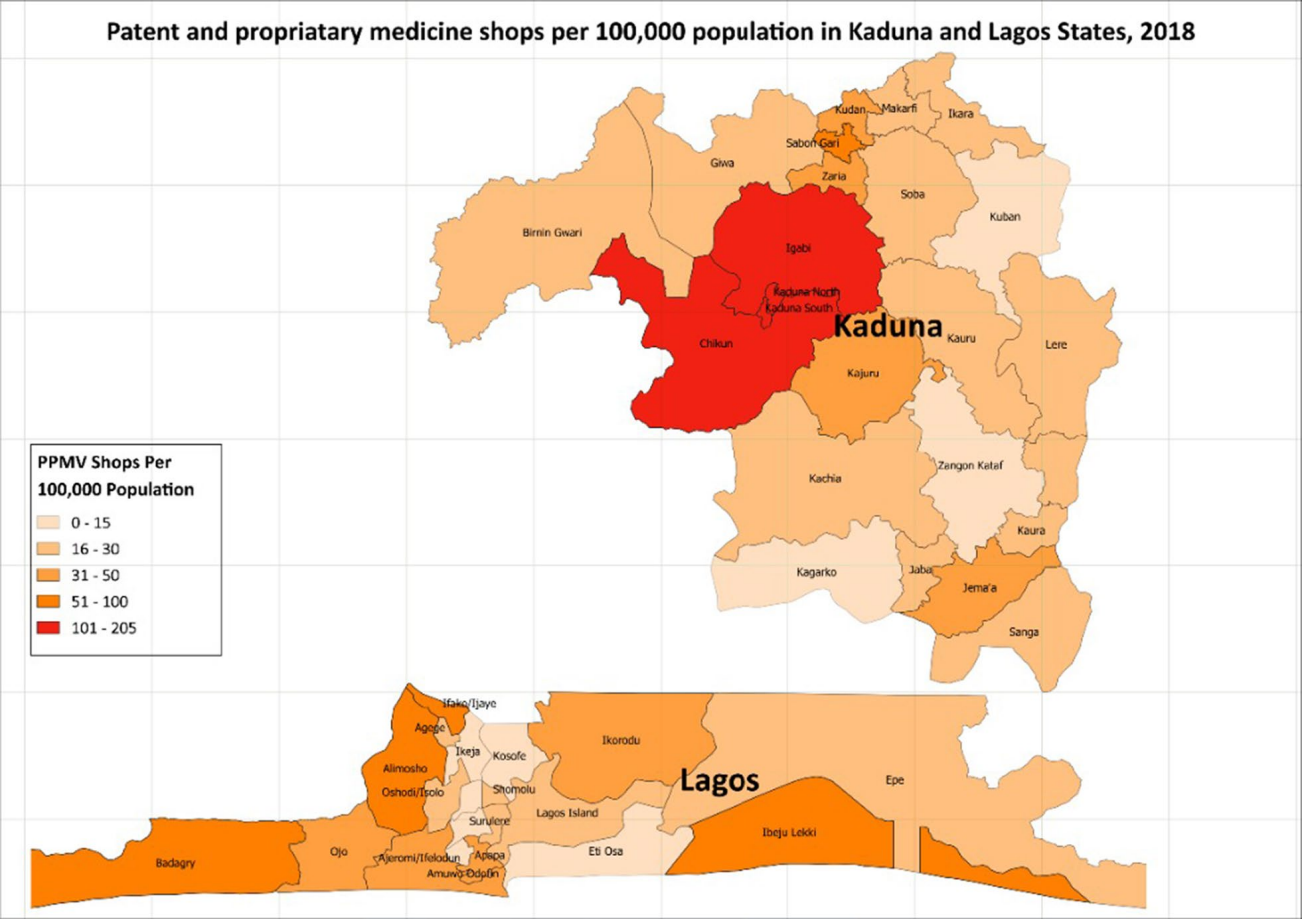
PPMV shops per 100,000 population in Kaduna and Lagos states

The results also show the number of medicine shops per 100,000 population in each of the LGAs. On average, there were 39 shops per 100,000 population in both states combined: 29 per 100,000 in the rural and 48 per 100,000 in the urban areas. More specifically, Kaduna State showed a higher percentage on the average; 46 shops per 100,000 population compared to 32 per 100,000 observed in Lagos State (maps shown in Fig. 3).

The assessment data also showed similar patterns with the census, with Kaduna (65.2%) and urban areas (84.4%) contributing largely to the sample. Table 3 shows additional characteristics of shops visited; we observed that 8 (81.6%) of every 10 medicine shops across these two states are open daily; only 8 shops reported opening for less than 6 days per week. The results also showed that 47% of the shops are relatively new businesses, opening within the last 5 years; about 10% (8.2%) opening within the same year as the study (2018). Registration status showed that more than half (56.1%) of all vendors were registered with the PCN, others registered with a national or local medicine dealers’ association; about one in every ten vendors (11.4%) were not registered with any association or regulatory body. Vendors operating in Lagos (Southern Nigeria) reported higher registration rates than their counterparts in Kaduna (Northern Nigeria); 83.2% compared to 41.6%.

**Table 3:** Facility assessment of patent and proprietary medicine shops in Kaduna and Lagos, 2018

Characteristics	Number (%) of shops (*N* = 874)
State	
Kaduna	570 (65.2)
Lagos	304 (34.8)
Location	
Urban	738 (84.4)
Rural	304 (15.6)
Number of days shop opens per week^a^	
≤ 5 days	8 (1.0)
6 days	140 (17.4)
7 days	657 (81.6)
Length of time business started^b^	
< 1 year	71 (8.2)
1–5 years	337 (38.8)
6–10 years	183 (21.1)
> 10 years	277 (31.9)
Registration	
Not registered with any association/regulatory body	100 (11.4)
National or local association of medicine vendors	284 (32.5)
Pharmacist Council of Nigeria	490 (56.1)
Number of client for family planning per day	
0	232 (28.5)
1	373 (45.8)
2	117 (14.4)
≥ 3	92 (11.3)
Ever attended training on family planning^c^	
Yes	219 (26.9)
FP product stocking practice^d^	
Any oral contraceptive	652 (86.8)
Injectable contraceptive	342 (45.5)
Emergency contraception	349 (46.5)
Intrauterine contraceptive device	10 (1.3)
Implant contraceptive	8 (1.1)
Open market as major source of FP products^c^	619 (76.0)

^a^Data available for only 805 shops

^b^Data available for only 868 shops

^c^Data available for only 814 shops

^d^Data available for only 751 shops

### FP stocking practices of PPMVs

On FP services/products being stocked by the medicine shops, about a quarter (25.7%) of vendors claimed to provide FP services to > 2 clients per week on the average; about the same number (26.9%) have had at least one person in the shop attend training on FP in the past. The majority (86.8%) of shops stocked at least one of the oral contraceptives, about half stocked injectable contraceptives (45.5%), and emergency contraceptives (46.5%). Only 18 shops stocked intrauterine devices or implant contraceptives. We also found that the majority (76.0%) of providers reported the open market as the major source of stocking these FP commodities; this however showed marked state differences. In Kaduna State, the open market accounted for the main source of supply of FP commodities, serving 95% of providers compared to less than half (42.1%) of shops in Lagos State.

### Association between medical training and FP product stocking, registration status and utilization of open drug markets

The bivariate analysis (shown in Table 4) found a significant association between medical training and all outcomes observed (product stocking of FP commodities, registration status, and supply from open drug market). Significant associations were also found between the length of time the PPMV business started and outcomes (except stocking of oral and emergency contraceptives). We also found a significant association between length of time business opened and medical training of vendor; findings show that a significantly larger number (56.3%) of newly opened PPMVs shops are manned by those who report medical training; those newly opened shops are 1.8 times more likely to be health trained compared to those opened > 5 years before the study. After controlling for confounders in the multivariate analysis (shown in Table 5), many of the associations remained significant (especially those relating to medical training of vendors). Consequently, there was a significant association between medical training and the likelihood of vendor stocking oral and injectable contraceptives. Also, there is a significantly reduced likelihood of medically trained vendors stocking from the open market compared to those not medically trained. Although there was an observed increased likelihood of newly opened PPMV shops to utilize the open market as the major means of stocking products, this relationship was not shown to be significant after adjusting for confounders.

**Table 4:** Bivariate analysis showing the association between medical training/newly PPMV outlets and product stocking, registration and source of FP products

Outcome	% of medicine vendors with any medical training			% of length of time PPMV business started		
	Yes	No	Sig	0–5 years	> 5 years	Sig
Product stocking^a^						
Any oral contraceptive	85.3	76.8	*	80.1	80.1	
Injectable contraceptive	65.2	27.1	**	48.9	35.8	**
Emergency contraception	48.9	39.0	*	42.3	43.4	
Registration						
National or local association of medicine vendors	44.9	31.6	**	41.0	33.0	*
Pharmacist Council of Nigeria	55.1	68.4	**	59.1	67.1	*
Source of FP products						
Open market	71.3	83.4	**	83.8	69.1	**
Open market (Kaduna)	94.8	95.3		95.9	93.8	
Open market (Lagos)	22.0	46.3	*	41.7	42.3	
Health training of vendor				56.3	43.7	**

*Sig.* significant association observed

*Significant at 5%; **significant at 1%, a multiple response possible (percentages are more than 100%)

**Table 5:** Multivariate analysis showing the association between medical training/newly PPMV outlets and product stocking, registration and source of FP products

Outcome	Model 1 (health training of vendor) OR (95% CI)^a^		Model 2 (PPMVs opened in ≤ 5 years) OR (95% CI)^b^	
	Crude	Adjusted	Crude	Adjusted
Product stocking				
Any oral contraceptive	1.75 (1.21–2.54)	2.20 (1.47–3.30)		
Injectable contraceptive	5.05 (3.73–6.84)	3.15 (2.17–4.58)	1.72 (1.30–2.28)	0.73 (0.50–1.06)
Emergency contraception	1.50 (1.13–1.99)	1.26 (0.92–1.74)		
Registration				
National or local association of medicine vendors	1.77 (1.31–2.38)	0.93 (0.65–1.33)	1.41 (1.05–1.90)	0.88 (0.62–1.25)
Pharmacist Council of Nigeria	0.57 (0.42–0.76)	1.08 (0.75–1.55)	0.71 (0.53–0.95)	1.14 (0.80–1.61)
Source of FP products				
Open market^*adj for state^	0.50 (0.30–0.84)	0.51 (0.30–0.87)	1.12 (0.73–1.72)	1.19 (0.77–1.85)
Health training of vendor			1.84 (1.39–2.42)	1.42 (1.04–1.94)

^a^Odds ratio (OR) with confidence intervals estimated using logistic regression. Reference category is vendors without medical training

^b^Odds ratio (OR) with confidence intervals estimated using logistic regression. Reference category is vendors who started operating more than 5 years before the study. Model 1 examines the association between medical training of vendor and registration, stocking and source of FP products while controlling for state, location (urban/rural), number of staff, number of FP clients, and length of time business started. Model 2 examines the association between length of time business started and registration, stocking and source of FP products while controlling for state, location (urban/rural), number of staff, number of FP clients, and medical training of vendor

Medical training of the vendor did not prove to be a significant predictor of registration status although some differences exist. Those who are medically trained seem to be more likely to register with the PCN than with national/local medicine dealers’ associations compare to those not medically trained. The same can be said for those who reported opening within 5 years of the study compared to PPMVs shops which have operated for longer periods.

## Discussion

The findings from this study, like the Liu et al. study among others, revealed that Patent Medicines shop were in abundance in the two states. With fewer health facilities and health posts opening across the country [29] and fewer pharmacies opening up (especially in rural and hard-to-reach areas) [14], PPMVs have remained the most widespread health structure across the 2 states; and across Northern and Southern Nigeria [18]. However, the study found a greater concentration of shops in the Northern state of Kaduna and especially in the urban areas; this is a slight deviation from the norm in many studies that PPMVs are mainly located in the rural and hard-to-reach areas [16, 22, 30].

As Nigeria strives to achieve Universal Health Coverage (UHC) and improve access to quality health services, the role of PPMVs has become more important. One of the domains of access listed in the UHC includes physical access to health services; this physical accessibility is dependent on the proximity of health facilities to potential clients [31]. In a recent study where the distribution of health facilities across Nigeria was documented, primary health facilities, more than other facilities were most prominent across the country (ranging 15.5 and 18.4 per 100,000 population in Southern and Northern states, respectively) [29]. The findings from our study showed that the distribution of PPMV shops in these regions more than doubled that of the most populous health facilities; ranking 32 and 46 per 100,000, respectively, in Southern state of Lagos and Northern state of Kaduna; this corroborates the findings of Liu et al. that PPMVs may be more accessible than healthcare facilities in many places across Nigeria. The figures in this study revealed a considerable increase from the figures shown in 2014 where there were 32 versus 24 per 100,000 in the Southern states and a similar 17 per 100,000 in the Northern States between PPMV shops and healthcare facilities [18]. The findings remained the same even when state comparison was done between this study and that the study by Makinde et al. In addition, the study found that PPMV shops have continued to grow progressively in the last 5 years, further increasing the margins between the numbers of PPMV shops and Health care facilities, and thus becoming the most proximal health facility for potential clients for different healthcare services.

Also, there is an emergence of PPMVs reporting formal medical training. These findings have also been observed by a few studies in the past [15, 18, 28] and are still relevant today. This suggests a deviation from the general definition of medicine vendors, who are characterized as being able to read and write [23] having completed mainly primary education and not having formal training in medicine and pharmacy [32, 33]; many of them were also thought to learn the trade through an apprenticeship program before opening their shops [16]. Our findings show that more than half of the vendors who reported medical training are relatively new in the business, opening within the last 5 years. This suggests the rise of vendors who may be able to deliver high-quality health services and complement the existing healthcare infrastructure [17] due to their formal training and previous experience (some being retired providers who worked at public/private health facilities across the country). Although the TSTS policy was launched within the last 5 years (in 2014), it is not likely this has any impact on the number or characteristics of new PPMVs coming into the business as until recently, tasks were not shifted to PPMVs regardless of their training and thus were not included in the TSTS policy, this study however suggests and supports evidence advocating for the expansion of the TSTS policy to include PPMVs [27, 34]. The findings from this study show a significant relationship between medical training of vendors and stocking of some FP commodities (especially injectable contraceptives) and with clients that patronize them for FP services every month (this supports findings elsewhere in Nigeria and Africa that PPMVs already offer injectable contraception and other services [27, 34]. This supports the evidence that PPMVs can support the formal health systems as these are services that they can provide, because of their training and the recent task-shifting and task-sharing policy of the country. A number of explanations are possible; firstly, retired health professionals do not necessarily exit the health labor force as some of them are actively engaged in health service provision in the private sector, such as medicine vending, thus should be taken account for comprehensive health workforce planning in the future. The study also shows some evidence of dual practice among health professionals, whereby some health professionals currently employed in public facilities are also engaged in the private sector by establishing their own medicines shops to supplement their incomes. However, how much income they earn and the implications of the dual practice on service provision and their ethical conduct were beyond the scope of the study. They could be explored further in an in-depth health labor market analysis. Unfortunately, some services being provided are currently prohibited within the PPMV scope [17, 26, 30, 32]. As many other studies have suggested, this study found that quality concerns may exist in the delivery of services by PPMVs as many of those interviewed stocked illegal commodities (beyond their legal scope of practice) and refused to register with the drug regulatory body (PCN) [16, 18, 30]. Despite the findings, studies have suggested that demand (especially women and adolescents) could be driving the stocking and sale of those categories of drugs especially when the drugs are not available in the formal health facilities [30, 35]. This, in addition to the geographic spread of the medicine vendors, has implications for the healthcare system in Nigeria and necessitates the expansion of more services (including FP) to PPMVs beyond their current scope of practice especially for those reporting formal medical training, as have been demonstrated in many pilot studies across Africa (including Nigeria) [22, 36–40]. This will be in line with the TSTS policy of the country, which calls for an increase in the capacity of community people to provide some reproductive, maternal, and child health services [18]. Strengthening the collaboration between this informal sector and the formal health sector may go a long way in improving the confidence of medicine vendors to legitimately stock drugs that they are capable of providing according to their qualification and improving referral for higher levels of care sought by the vast population that patronize them.

The findings of the study also show that health training of PPMV and length of time in which the PPMV business opened may influence registration status with the PCN, although this was not found to be significant after adjusting for the state, location, and the number of FP clients. There is a possibility that newly opened shops with medically trained vendors are more likely than others to register with the PCN, suggesting a possible improvement in the relationship between PCN and the association of patent vendors in the last 3 years, this finding show that health trained vendors may be more likely to follow regulatory and quality assurance guidelines; this corroborates the findings found by other investigators [18, 30]. This also suggests that newly opened vendors may be willing to register with the PCN at the start, but the influence of older PPMVs and the majority who are without health training may further drag new PPMVs into not desiring to register annually with the regulator, but rather with the professional associations, or not registering at all altogether and operating illegally. As noted by several studies, the medicine dealers’ associations are perceived to be more beneficial to the vendors than PCN for several reasons ranging from providing them with protection against law enforcement agents to peer-to-peer mentoring and oversight functions (especially with preventing illegal drug sellers and monitoring of the sale of drugs) to its members through a taskforce; they are generally more acceptable by the medicine vendors, evidenced by the nearly 4 out of every 10 vendors, who did not register with the PCN in this study [24, 30]. There is thus a need for a collaborative framework between the associations and PCN to improve registration rates and perception of medicine vendors about the roles of PCN from an antagonistic standpoint. PCN needs to be seen to perform more supportive roles to foster trust and respect, which could lead to a suitable accreditation system, an opportunity for professionals to expand their scope through continuous learning, and ultimately have the drug list expanded to fit the realities of the sector. This may result in more health trained vendors joining the business and offering a great opportunity to further improve the health of the populace. The presence and availability of a ready source of healthcare in the private sector at community level is important for scaling up access to primary health care in rural areas and urban slums across the country. Currently, the IntegratE project is working with the Federal Ministry of Health and its parastatals including the Food and Drugs Department and the Pharmacists Council of Nigeria on a tiered accreditation system for PPMVs. This is currently being pretested for feasibility.

Lastly, our findings support the claim of many studies that many vendors utilize the open market as the major source of supplying health products [17, 18, 41, 42]. The National Drug distribution guideline, however, has as part of its core deliverables, the elimination of the dominance of unregulated drug markets in major cities of Nigeria [43, 44]; the findings from our study show that these markets still supply the majority of the vendors (especially in the Northern part of the country). In the southern part of the country, reliance on open markets is common among those without health qualifications and in the rural areas (where mega dealers/wholesalers are few). Drug distribution requires efficient supply chain systems and appropriate regulation to ensure that the medicines that reach the consumer are in their intended qualitative state, supported with the required infrastructure to ensure rational use; this, however, is not the case across both states as vendors still source for drugs in these markets where drugs are peddled without caution. For the most part, only the public and few private health facilities are catered for by the national drug distribution centers (MDDC and SDDC) [45].

This study has several limitations. Chiefly, by combining the results of two studies conducted at different times, there is a possibility of missing some key characteristics from shops not included in both studies, especially as the second study (facility assessment), focused on PPMVs in proposed implementation LGAs. Also, recall bias is a possibility with this study type as some of the variables self-reported by PPMVs could not be verified in both studies, only GPS coordinates, licensing status with the PCN, and product stocking could be independently verified.

## Conclusion

PPMVs have continued to grow progressively in the last 5 years, becoming the most proximal health facility for potential clients for different healthcare services (especially FP services) across both Northern and Southern Nigeria. They now comprise a considerable mass of medically trained personnel, able to deliver high-quality health services and complement existing healthcare infrastructure if trained. However, restrictions on what is possible within the PPMV premise and lack of access to quality drugs/commodities have resulted in some poor practices among PPMVs. There is therefore a need to identify, train, and provide innovative means of improving access to quality-assured products for this group of health workers. This may significantly reduce the stocking of inadequate or substandard medicines, reduce the stock-out of key health commodities, and further improve the quality of services provided by these providers and ultimately health outcome of the populace.

## Data Availability

The datasets used and/or analyzed during the current study are available from the corresponding author on reasonable request.

## Abbreviations

APML: Approved Patent Medicine List
CI: Confidence interval
CSPro: Census and Survey Processing System
DMPA-Sc: Subcutaneous depomedroxyprogesterone acetate
FP: Family planning
GPS: Global Positioning System
LGA: Local Government Areas
LMICs: Low- and middle-income countries
MDDC: Mega Drug Distribution Centre
OR: Odds ratio
PCN: Pharmacists Council of Nigeria
PPMV: Patent and Proprietary Medicine Vendors
SDDC: State Drug Distribution Centre
TSTS: Task-shifting and task-sharing
UHC: Universal Health Coverage
WHO: World Health Organization
